# Subclinical Elevation of Plasma C-Reactive Protein and Illusions/Hallucinations in Subjects with Parkinson’s Disease: Case–control Study

**DOI:** 10.1371/journal.pone.0085886

**Published:** 2014-01-31

**Authors:** Hideyuki Sawada, Tomoko Oeda, Atsushi Umemura, Satoshi Tomita, Ryutaro Hayashi, Masayuki Kohsaka, Kenji Yamamoto, Shinji Sudoh, Hiroshi Sugiyama

**Affiliations:** Clinical Research Center and Department of Neurology, National Hospital of Utano, Kyoto, Japan; Chiba University Center for Forensic Mental Health, Japan

## Abstract

**Background:**

Though infections are associated with psychotic symptoms, whether or not subclinical inflammation is associated with hallucinations is not known in Parkinson’s disease (PD).

**Purpose:**

To investigate the association of illusions/hallucinations and plasma CRP levels in PD patients without symptomatic infections.

**Methods:**

PD patients not diagnosed as having infections were assessed for illusions and hallucinations using the Parkinson Psychosis Questionnaire (PPQ). It comprises four-domain questions: PPQ-A for sleep problems, PPQ-B for hallucinations/illusions, PPQ-C for delusions, and PPQ-D for disorientation. Assigning patients with ≥1 points in the PPQ-B score to be cases and others as controls, the association of hallucinations/illusions and clinical features (age, sex, duration of PD, Unified Parkinson’s Disease Rating Scale part 3 (UPDRS-3), Mini-Mental State Examination (MMSE) score, sleep disturbance (PPQ-A score) as well as daily doses of L-Dopa, dopamine agonists, amantadine, and selegiline) were analyzed using a case–control design.

**Results:**

A total of 111 patients were examined and plasma CRP levels were <0.1–6.0 mg/L. Hallucinations or illusions were detected in 28 (25.2%). There were significant differences in age, UPDRS-3 score, MMSE score, PPQ-A, daily doses of L-Dopa and dopamine agonists and plasma CRP levels between cases and controls. A multivariate logistic regression model revealed that UPDRS-3 scores and plasma CRP levels were significantly associated with hallucinations/illusions with an adjusted odds ratio of 1.96 (95% confidence interval (CI) 1.20–3.20) per 10 points and 1.57 (95% confidence interval 1.13–2.16) per two-fold, respectively. Dividing patients into thirds by CRP levels (≤0.2, 0.3–0.6, ≥0.7 mg/L), the prevalence of hallucinations/illusions was 13.2%, 21.6%, and 41.7%, in the bottom-, middle-, and top-thirds, respectively (for trend p = 0.012).

**Conclusions:**

Subclinical elevation of plasma CRP levels was associated with hallucinations or illusions after adjustment for motor disability, suggesting that subclinical elevations of CRP levels might be an independent risk for hallucinations/illusions.

## Introduction

Parkinson’s disease (PD) is characterized clinically by bradykinesia, muscular rigidity and tremor, and pathologically by dopaminergic neurodegeneration. The motor symptoms are caused by striatal dopamine deficiency and are relieved by dopamine replacement therapy. However, non-motor symptoms such as autonomic failure, cognitive decline and psychosis are not improved by dopaminergic replacement therapy [1], [2]. Among non-motor symptoms, psychosis (e.g., hallucinations, delusions) is important because it can be a heavy burden on family or caregivers [3] or associated with poor outcomes (including persistent psychosis, placement in a nursing home or death) [4]–[7]. Hallucinations range from very mild forms such as “de passage hallucination” or sense of presence, to vivid hallucinations without insight of patients [7]–[9]. Mild hallucinatory experiences cannot be detected in routine neurological examinations for motor symptoms. They can be overlooked because patients may not complain of hallucinations, especially if they are mild and patient insights are preserved. Because of an inconsistent definition of psychosis, the prevalence range has varied in reports [8], [10]–[12].

Cognitive decline [10], [13]–[16], severities of PD [13], [16], or sleep disturbances [12], [14], [17], and a history of hallucinations [18] are predictive of hallucinations. However, mild hallucinatory experiences often occur transiently or intermittently, and improve spontaneously [19], suggesting that they could be associated with the transient medical conditions of patients.

In this cross-sectional study, we evaluated psychosis in patients with PD using the Parkinson Psychosis Questionnaire (PPQ). PPQ consists of four domains: sleep disturbances (A), hallucinations or illusions (B), delusions (C), and orientations (D) [20]. The PPQ has been designed to detect the early symptoms of psychosis, and the scores correlate highly with the Brief Psychiatric Rating Scale.

Though serious medical conditions (including systemic inflammations) are associated with psychotic symptoms [21], whether subclinical inflammation is a risk factor for psychosis in PD patients it has not been elucidated. C-reactive protein (CRP) is synthesized in the liver and is a peripheral marker of systemic inflammation but is also associated with atherosclerosis and cardiovascular diseases (including coronary insufficiency) [22], [23]. In addition, CRP has been reported to be associated with risk of PD [24], [25], and neuro-inflammation might be associated with the neurodegenerative process of PD [26]. The purpose of the present study was to investigate the associations of the clinical features and psychosis, especially hallucinations/illusion. Mild hallucinations/illusions are common in PD patients, but delusions are not common (especially in the early stages) [11]. In this context, we focused on the association of plasma CRP levels and hallucinations/illusions. Therefore, we designated patients with PPQ-B ≥1 as “cases” and the others as “controls.”

## Methods

### Study Design

We conducted a cross-sectional case-control study to investigate the association of hallucinations/illusions and clinical features: age, sex, PD duration, motor disability, cognitive function, medications for PD, sleep disturbance, serum albumin concentrations, and plasma CRP levels.

### Ethical Approval

This study was approved by the Bioethics Committee of Utano National Hospital. According to the Bioethics Committee, informed consent was not needed in patients with PD, because the study was a retrospective observation study and data were analyzed anonymously. To obtain the data of healthy controls and patients with Alzheimer disease, the study protocol was revised and approved by the Bioethics Committee, and in healthy controls and patients with Alzheimer disease, written informed consent was obtained because the data were collected for this study. In patients with Alzheimer disease who have a compromised capacity to content, their family (spouses, sons, or daughter) were also explained the purpose and methods of the study, and the family gave the written consent on behalf of the patient.

### Subjects

#### PD patients

We enrolled patients with PD who were diagnosed as having PD and not diagnosed as having infections. Study patients were recruited from a screening list for a clinical trial conducted in the National Hospital of Utano (Clinical Trial Registration Number: UMIN000005403) [27]. The diagnosis of PD was made according to step 1 and step 2 for the diagnostic criteria for PD in the United Kingdom. Patients were not selected on the basis of having a history of psychosis. Those who were diagnosed as having infections such as pneumonia, bronchitis, urinary cystitis, pyelonephritis or who were treated with antibiotics or with corticosteroids were excluded. Patients who were prescribed anti-psychotic medications were also excluded.

#### Healthy controls and patients with alzheimer disease

Spouses of PD patients were recruited and those who were healthy were enrolled as healthy controls. In addition, patients who were diagnosed as probable Alzheimer’s disease dementia according to criteria from the National Institute on Aging-Alzheimer’s Association workgroup were also enrolled [28]. Those who were diagnosed as having infections such as pneumonia, bronchitis, urinary cystitis, pyelonephritis or who were treated with antibiotics or with corticosteroids were excluded. Patients who were prescribed anti-psychotic medications were also excluded.

### Evaluation of Psychosis, Cognitive Function, Extrapyramidal Signs, Sleep Disturbance, and Concurrent Medications

The PPQ was used to evaluate hallucinations or illusions [20]. Scoring of PPQ was described elsewhere [27]. Briefly, 4 questions about illusions, visual hallucinations, auditory hallucinations, and tactile hallucinations were evaluated with regard to frequency (0, none; 1, only during the night; 2, during the night and the day; 3, almost every day and night) and severity (0, none; 1, with retained insight; 2, no full insight; 3, lacking insight) by PPQ-B questions. The scores were calculated by multiplying the frequency and the severity (range: 0–9). Sleep disturbance, including restlessness during the night, vivid dreams and nightmares, were assessed by PPQ-A questions with respect to frequency (0, none; 1, up to once per week; 2, several times per week, but not daily; 3, once or more times per day) and severity (0, none; 1, not affecting patient’s or caregiver’s wellbeing; 2, moderately affecting wellbeing; 3, severely affecting wellbeing). The scores were calculated by multiplying the frequency and severity (range: 0–9). The Mini Mental Status Examination (MMSE) and Unified Parkinson’s Disease Rating Scale (UPDRS-3) were used to evaluate the cognitive function and motor severity of PD. In patients with “on–off” or “wearing-off” phenomena, the UPDRS-3 was used in the “on period.” The daily doses of L-Dopa, entacapone, dopamine agonists (ropinirole, pramipexole, pergolide, and cabergoline), amantadine and selegiline were collected as concurrent medications for PD. Blood samples were obtained for assessment of levels of CRP and albumin as well as white blood cell (WBC) count. Experience of hallucinations or illusions during the previous 28 days is checked in the PPQ, so blood samples that were obtained within 28 days before the day of the PPQ were collected if the data of blood samples of the day of the PPQ were missed.

### Definitions of Cases and Controls

To investigate clinical factors associated, patients with 1 or more PPQ-B scores were regarded as cases (with hallucinations or illusions), and other patients were regarded as controls (without hallucinations or illusions).

### Data Collation

Age, sex, duration of PD, modified Hoehn–Yahr stage (H–Y), UPDRS-3, MMSE, dose of dopaminergic replacement therapy, and data from blood samples were collated. History of psychosis and initial symptoms (tremor-dominant or others) were also collected. The dopaminergic medications were L-Dopa, dopamine agonists, selegiline, amantadine, and entacapone. The daily dose of Dopa was calculated using the formula:
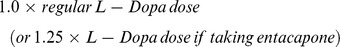



The dose of dopamine agonists was calculated as the L-Dopa equivalent dose (LDED) according to the formula:
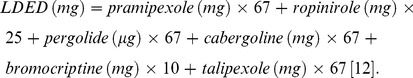



Blood sampling was done the day when the PPQ was used. However, data obtained ≤28 days before the PPQ could be incorporated if the sample could not be assessed the day of the PPQ. Plasma CRP (mg/L), WBC counts (per mm^3^), and albumin (mg/dL) were collected. CRP was assayed using a high-sensitivity method.

### Variables

Age, duration of PD, MMSE, UPDRS-3, as well as the daily dose of L-dopa, dopamine agonist, amantadine, and selegiline were regarded as scale variables. H–Y stage was the ordinal variable and was treated as a categorized variable (H-Y 1–3 *versus* 4–5). Because of non-Gaussian distributions, for comparison of daily doses of dopamine agonists, amantadine, and selegiline as well as CRP levels, non-parametric analyses were adopted. In the logistic regression model, CRP was transformed to log_2_ CRP because it is known that log CRP is distributed normally [23].

To investigate multicollinearity between scale predictable variables, scattered plots of the correlation matrix were obtained. Then, Spearman’s correlation coefficient was calculated to exclude variables with a correlation coefficient greater than 0.8 or lesser than −0.8.

### Statistical Analyses

The standard deviation of log_2_CRP was estimated 1.59 [23]. Mild hallucinations/illusions that (defined as PPQ-B score ≥1 point) were found in ≈ 30% patients in our preliminary study [29]. Therefore, we assumed that the proportion of cases and controls was 30% and 70%, respectively. Assuming the CRP level in cases to be twice that in controls, the effect size was 1.0 in log_2_CRP. The sample size was calculated to be 94 (66 controls and 28 cases) on the conditions that α = 0.05 (bilateral) and β = 0.2 (the power 80%).

Age, duration, MMSE, daily dose of L-dopa, WBC count, or albumin levels were compared between cases and controls using the Student’s *t*-test because Gaussian distribution was assumed in these scale variables. Scale variables with non-Gaussian distributions (doses of dopamine agonists, amantadine and selegiline, as well as CRP levels) were compared using a non-parametric test. Categorical variables such as sex or H–Y stage (1–3 *versus* 4–5) were compared using the chi-square test. The relative risk of hallucinations/illusions was estimated as an odds ratio (OR) using logistic regression models. Variables that were different between cases and control with p<0.1 were incorporated in the logistic regression models. ORs were calculated in bivariate regression models and a multivariate regression model. Furthermore, to identify suitable predictable variables, predictable variables were selected using a backward likelihood ratio test. In a multivariable logistic regression model, fitness was evaluated by plotting the observed probability of cases (with mild hallucinations/illusions) and that estimated by a logistic model and, in addition, tested by Hosmer-Lemeshow method. P values of less than 0.05 were considered statistically significant. Statistical analyses were performed using the statistical software program IBM SPSS version 21.

## Results

The PPQ was employed in 121 patients with PD. Analyses were restricted in 111 patients because information about CRP levels during 28 days was not available in 10 patients. Complete information about age, sex, duration of PD, H–Y stage, UPDRS-3 score, MMSE, daily dose of medications for PD, plasma level of CRP, albumin level, and WBC count was obtained. Information about the history of psychosis and initial PD symptoms were obtained in 100 and 107 patients, respectively.

**Figure 1:**
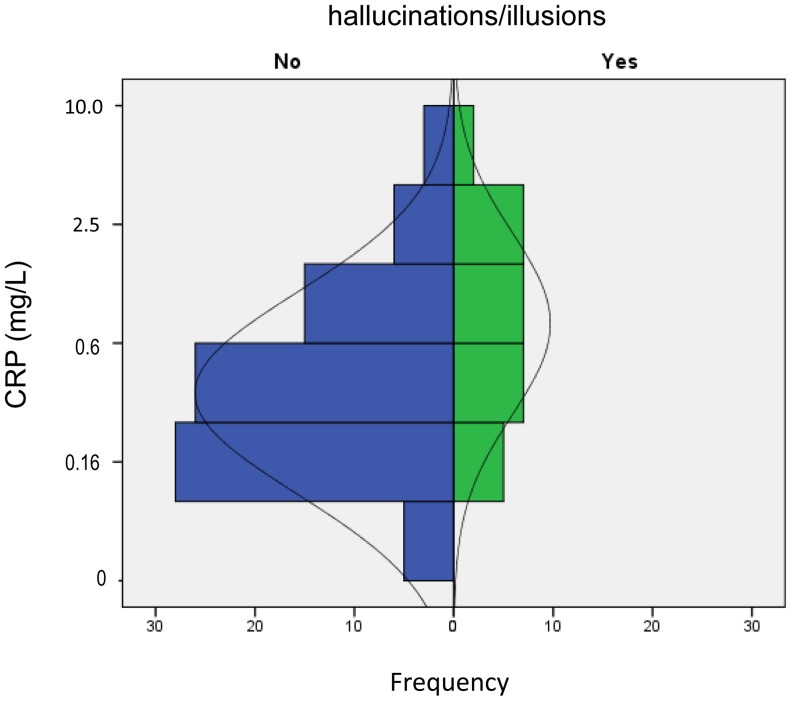
Pyramid histogram of CRP levels in cases (green) and controls (blue). Plasma CRP levels (expressed as log_2_CRP) were distributed as a bell-shape. They were shifted upward in cases compared with those in controls.

The PPQ-B score was 0 in 83 patients and they were assigned as “controls,” and 28 patients (1 point in 9 patients, 2 points in 12 patients, 3 points in 4 patients, 4 points in 1 patient, and 6 points in 2 patients) were assigned as “cases”. The PPQ-C score (delusions) was 0 in 105 patients, and 6 patients with PPQ-C ≥1 point had PPQ-B ≥2 points. The relationship between PPB-B scores and PPQ-C scores was shown in **Table S1** and psychotic symptoms in 28 cases were listed in **Table S2**. The clinical characteristics of cases and controls are shown in **Table 1**. Cases were older than controls, UPDRS-3 scores were higher, and MMSE scores lower in cases with statistical significance. In cases, 50% of patients had a history of psychosis, whereas in controls only 24% had a history of psychosis. There was no difference in initial symptoms (tremor-dominant or others) between cases and controls. The PPQ-A score was higher in cases. The daily dose of dopamine agonist was lower, and that of L-dopa higher, in cases. Plasma levels of CRP were higher in cases. P-P plots and Q-Q plots demonstrated that the distribution of CRP levels was non-Gaussian but that, after transformation to logarithms, log_2_CRP levels were distributed in an almost Gaussian manner (**Figure S1**). Therefore, according to the assumption described in the Methods section, CRP levels were transformed to log_2_CRP levels in further analyses. The distribution of patients according to CRP levels is demonstrated in cases and controls in **Figure 1**.

**Table 1:** Participant clinical features by hallucinations.

		Hallucinations		No hallucinations		*p*
n		28		83		
Age (Y)	mean (SD)	73.1	(7.1)	68.6	(7.7)	0.006
Sex (Male)	n (%)	13	(46%)	33	(40%)	0.7
Duration of PD (Y)	mean (SD)	9.0	(4.2)	7.7	(4.6)	0.15
history of psychosis	Yes, n (%)	11	(52%)	19	(24%)	0.02
	No, n (%)	10	(48%)	60	(76%)	
Initial symptoms	Tremor, n (%)	11	(46%)	36	(43%)	1.00
	Others, n (%)	13	(54%)	47	(57%)	
modified H-Y	4–5, n (%)	14	(50%)	26	(31%)	0.11
	1–3, n (%)	14	(50%)	57	(69%)	
UPDRS-3	median (IQR)	27	(20–39)	20	(14–26)	0.002
MMSE	median (IQR)	26	(24–29)	29	(26–30)	0.004
PPQ-A (sleep disorder)	median (IQR)	1	(0–2)	0	(0–1)	0.046
L-Dopa dose (mg/day)	mean (SD)	493.8	(184.7)	414.8	(173.9)	0.09
DA agonist (LDED mg/day)	median (IQR)	25.1	(0–134)	100	(0–168)	0.06
selegiline (mg/day)	median (IQR)	0.0	(0.0–3.8)	0.0	(0.0–5.0)	0.50
amantadine (mg/day)	median (range)	0.0	(0.0–50)	0.0	(0.0–0.0)	0.23
plasma CRP (mg/L)	median (IQR)	0.8	(0.3–1.8)	0.4	(0.1–0.8)	0.003
white blood cell count	mean (SD)	5,568	(1,166)	5,233	(1,419)	0.26
albumin (mg/dL)	mean (SD)	4.0	(0.3)	4.1	(0.3)	0.09

Statistical analysis was performed as follows; t-test for age, duration, UPDRS–3, MMSE, Dopa dose, white blood cell count, and albumin. Chi-square test for modified H-Y stage. Mann-Whitney U test for PPQ-A score, DA agonist, selegiline, amantadine and plasma CRP because of non-Gaussian distributions.

**Figure 2:**
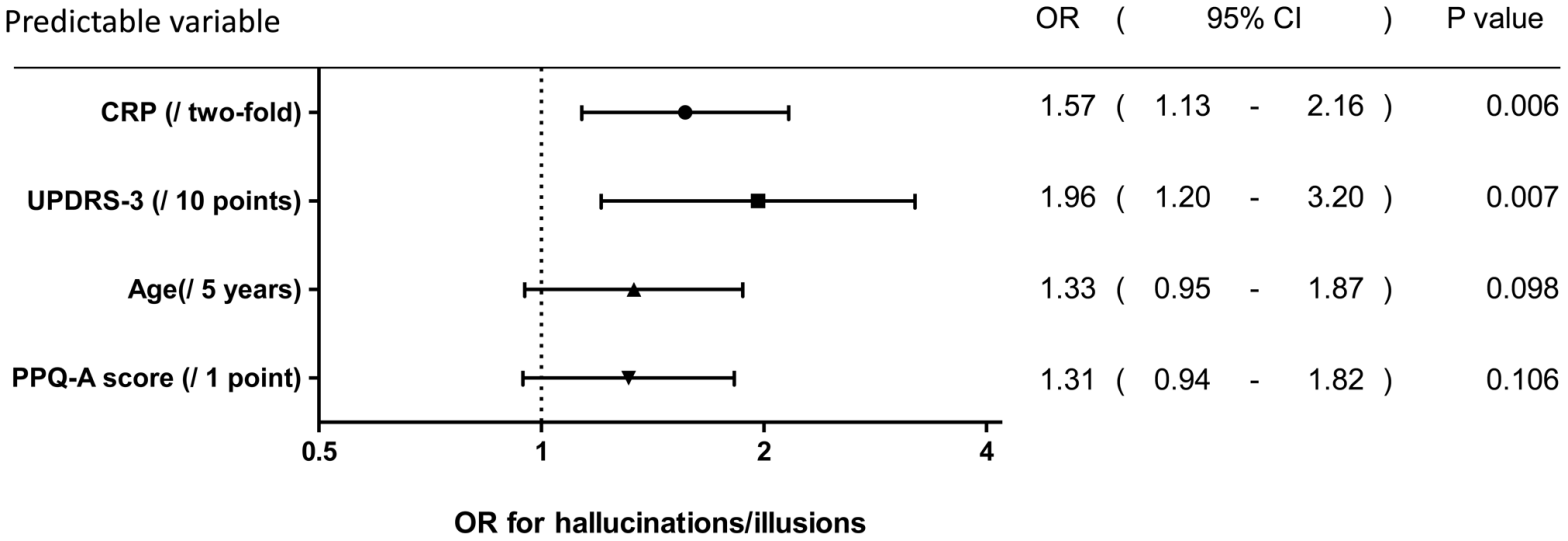
Relative risk of hallucinations/illusions estimated as odds ratios (ORs) using a multi-variable regression model. Predictable variables were selected using a backward likelihood test, and plasma level of CRP, age, and MMSE score incorporated into the model.

**Figure 3:**
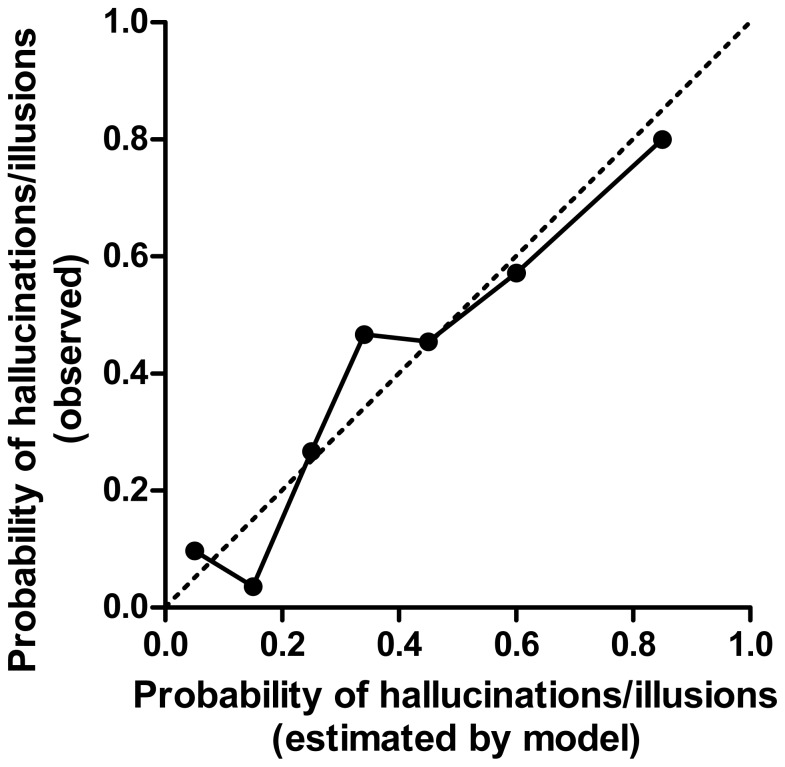
Observed and estimated probability of hallucinations/illusions. Line demonstrates the relationship between the observed probability of hallucinations/illusions and that estimated in the logistic regression model incorporating plasma CRP, age and MMSE as predictable variables.

Scattered plots of scale predictable variables did not show multicollinearity between them (**Figure S2**). In addition, there were no scale variables with a correlation coefficient great than 0.5 or lesser than −0.5, so there was no multicollinearity between the scale variables (**Table S3**). Among these factors, variables with p<0.1 (i.e., CRP levels, UPDRS-3 score, MMSE score, PPQ-A score, age, daily dose of L-dopa and dopamine agonists, and albumin concentration) were assessed, and the associations of these factors and hallucinations/illusions were estimated using logistic regression models. ORs were calculated using bivariate logistic models and multivariate logistic models (**Table 2**). Bivariate logistic models demonstrated that all of the predictable variables were associated with hallucinations/illusions. The multivariate logistic regression model demonstrated that daily doses of L-dopa and dopamine agonists as well as albumin concentrations were not associated with hallucinations/illusions. UPDRS-3 score and CRP levels were highly and significantly associated with hallucinations/illusions. The OR of log_2_CRP was 1.45 (95% confidence interval (CI) 1.03–2.04). To identify the most appropriate predictable variable, variables were selected statistically using the backward likelihood ratio test. As a result, plasma CRP levels, UPDRS-3 score, age and PPQ-A score were selected. After adjustment of these variables, the OR of log_2_CRP was 1.57 (95% CI 1.13–2.16) with statistical significance in this model (**Figure 2**). To evaluate the fitness of the logistic regression model, the relationship between the observed probability of cases and estimated probability was plotted (Figure 3) and demonstrated the fitness of the model. The chi-square of the Hosmer–Lemeshow test was 6.81 and *p* was 0.557, suggesting good fitting of the model.

**Table 2:** Relative risk of hallucinations.

	Bivariate Odds Ratios			Multivariate adjusted Odds Ratios		
Factors	ORs	(95% CI)	*p*	ORs	(95% CI)	*p*
plasma CRP, per two-fold	1.53	(1.16–2.02)	0.003	1.50	(1.08–2.01)	0.016
UPDRS-3, per 10 points	2.05	(1.33–3.17)	0.001	1.91	(1.10–3.32)	0.022
MMSE, per 5 point	0.28	(0.11–0.69)	0.006	0.54	(0.18–1.64)	0.28
Age, per 5 years	1.49	(1.09–2.04)	0.012	1.33	(0.89–2.01)	0.16
Dopa, per 100 mg	1.24	(0.97–1.60)	0.091	1.13	(0.81–1.59)	0.47
DA agonist, per 100 mg(LDED)	0.62	(0.4–1.04)	0.072	1.24	(0.59–2.64)	0.57
Albumin, per 0.1 mg/dl	0.90	(0.78–1.04)	0.146	1.02	(0.86–1.22)	0.79
PPQ-A score	1.29	(0.93–1.77)	0.125	1.30	(0.90–1.88)	0.16

According to the plasma level of CRP, patients were divided into three groups: bottom-third, middle- and top-third (Table 3). There was a significant difference in the prevalence of patients with hallucinations or illusions (for trend, p = 0.035). MMSE score was lowest in the bottom-third and highest in the top-third (p = 0.01). Age was lowest in the bottom-third and highest in the top third (p = 0.0498). In contrast, there were no significant differences in UPDRS-3 score, daily doses of L-dopa and dopamine agonists, or PPQ-A score. These data suggested that plasma levels of CRP were associated with MMSE score and age, as well as with the prevalence of hallucinations/illusions. To confirm the result, the association of CRP (log_2_CRP) and hallucinations/illusions was estimated in another logistic regression model with adjustment for MMSE score and age, the OR of log2 CRP level was 1.43 (95% CI; 1.06–1.93, p = 0.019).

**Table 3:** Prevalence of hallucinations by thirds of plasma CRP.

		Plasma CRP						
		bottom third		middle third		top third		
		<0.2 mg/L		0.3–0.6 mg/L		0.7 mg/L<		*p*
Hallucinations	Yes, n (%)	5	(13.2%)	8	(21.6%)	15	(41.7%)	0.005
	No, n (%)	33	(86.8%)	29	(78.4%)	21	(58.3%)	
UPDRS-3	mean (SD)	21.2	(10.1)	23.6	(11.4)	23.9	(10.2)	0.44
MMSE	mean (SD)	28.3	(2.0)	27.7	(2.0)	26.4	(3.5)	0.002
Age (Y)	mean (SD)	67.2	(7.8)	70.8	(7.3)	71.1	(7.9)	0.05
DA agonist (LDED, mg/day)	median (IQR)	83.5	(0–168)	67.0	(0–200)	71.0	(0–134)	0.70
L-Dopa (mg/day)	mean (SD)	435.5	(183.2)	412.8	(185.7)	456.3	(170.0)	0.59
PPQ-A score	median (IQR)	1	(0–2)	0	(0–1)	1	(0–2)	0.09

*p* was calculated using Chi-square test for hallucinations (p for trend), ANOVA for UPDRS-3, MMSE, age, L-Dopa, Kruskal-Wallis test for DA agonist and PPQ-A score.

To investigate whether or not the CRP levels are associated with hallucinations/illusions in healthy elderly people or patients with Alzheimer disease, data of PPQ and plasma CRP were obtained in 47 healthy elder people and 6 patients with Alzheimer disease. Mean (standard deviation) of age was 69.1 (7.1) in healthy controls and 78.8 (4.9) Alzheimer disease patients. The scores of PPQ-B and PPQ-C were 0 in all of them, and nobody had hallucinations, illusions or delusions. The level of plasma CRP (median (IQR)) was 0.50 mg/L (0.30–1.20 mg/L) and 0.45 mg/L (0.10–0.6 mg/L), respectively. The distribution of CRP in healthy controls was very similar to that in PD patients, and none of these subjects experienced hallucinations/illusions (**Figure S3**). There was no statistically significant difference in log_2_CRP between PD patients and healthy elders.

## Discussion

In the present study, patients who were not diagnosed as having inflammation were enrolled, and plasma levels of CRP were <0.1–6.0 mg/L (i.e., in the normal range). Hallucinations and illusions were evaluated by PPQ-B score, and patients with ≥1 point of the PPQ-B score were regarded as having hallucinations/illusion. Patients with delusions (PPQ-C >1 point) had PPQ-B >1 point, and therefore, they were included in cases. Hallucinations or illusions with 1 point of the PPQ-B are those which have retained insight or which occur in the night and, therefore, very mild forms of hallucinations or illusions were involved in the cases in the present study. The design was a cross-sectional observation study, which is suitable to assess the relationship between hallucinations/illusions and clinical factors. However, psychotic symptoms are considered to be the cumulative results of various neurobiological and psychosocial factors, and these factors could not be evaluated fully in a cross-sectional study. In spite of this limitation of the study, plasma CRP was statistically associated with hallucinations/illusions. However, hallucinations/illusions were also associated with the motor disability of PD, results which are very consistent with previous reports [30]. In patients with advanced PD, due to motor deteriorations, respiratory infections are a common complication. Therefore, plasma levels of CRP might be associated with motor disturbances. High levels of CRP have been reported to be associated with the development of dementia in patients with mild cognitive impairment [31], so cognitive function might also be associated with CRP levels. In this context, plasma levels of CRP might be confounded with motor disability and cognitive function. To confirm such possible confounding, we divided patients into “thirds” according CRP levels. The prevalence of patients with hallucinations/illusions increased in accordance of elevation of CRP levels (**Table 3**). Unexpectedly, there was no significant difference in UPDRS-3 score in the thirds. This finding suggested that elevation of CRP levels was not due to motor disturbances. In contrast, there was a significant trend in MMSE score and CRP level; the MMSE score was highest in the bottom-third and lowest in the top-third. In this context, our results suggested the possible confounding relationship between the MMSE score and plasma levels of CRP.

However, after adjustment of the UPDRS-3 score, MMSE score, age, daily doses of L-dopa and dopamine agonists, albumin concentration, and PPQ-A score, the OR of CPR levels was 1.45 (per twice, 95% CI 1.03–2.04) and was significantly different from controls. These data suggest that elevation of plasma levels of CRP is associated with hallucinations/illusions and that the association is independent of motor disability or cognitive decline.

Several studies have suggested that levels of pro-inflammatory cytokines (including interleukin-6) are elevated in PD and are pointed to be associated with non-motor symptoms [32]–[34]. In PD brains, microglia are thought to be activated and neuro-inflammation (including microglial activation) involved in the neurodegenerative process [35]–[37]. Hassin-Baer and colleagues investigated the association of CRP levels and non-motor symptoms in PD, and demonstrated that CRP levels were associated with depression but not with psychosis. Though the reason for the discrepancy between their negative result and that in the current study is not known, the distribution of CRP levels was very different between their cohort and the cohort in the present study [38]. Recent studies have suggested that mild systemic inflammation could influence brain function in Alzheimer disease [39] or Schizophrenia [40]. In PD as well, mild hallucinations/illusions could be elicited by subclinical inflammation. The permeability of the blood brain barrier is increased by CRP [41], [42], and in the conditions where CRP is elevated, cytokines might have effects in the brain. However, it is unknown whether or not subclinical elevation of plasma levels of CRP increases the permeability. Because of no differences in CRP levels between PD patients and healthy controls, subclinical elevation of CRP influences brain function in PD patients but not in healthy elders.

## Supporting Information

Figure S1 **Normal P-P plots and normal Q-Q plots of plasma CRP (A, B) and log_2_CRP (C, D).** Normal P-P plots and normal Q-Q plots of plasma CRP were shown in A and B, respectively, suggesting that the distribution of CRP was not Gaussian. In contrast, Normal P-P plots and normal Q-Q plots of log_2_CRP suggested that the distribution log_2_CRP was Gaussian (C, D).
(TIF)

Figure S2 **Matrix scattered plots of predictable variables.** Scale variables (age, duration of PD, UPDRS-3 scores, MMSE scores, and daily dose of Dopa, dopamine agonists, amantadine, and selegiline) were plotted to check their multicollinearity. There was no multicollinearity in these scale variables.
(PDF)

Figure S3 **Histogram of log_2_CRP in PD patients and healthy controls.** Log2CRP was distributed normally both in PD patients (blue, n = 111) and healthy elderly controls (n = 47, green). The distribution was very similar in PD patients and healthy controls.
(PDF)

Table S1 **Relationship between PPQ-B (hallucinations/illusions) and PPQ-C (delusions).**
(PDF)

Table S2 **Psychosis in 28 cases.**
(PDF)

Table S3 **Spearman’s correlation coefficient between scale predictable variables.**
(PDF)
