# Supplementary figures and images for: Subclinical Elevation of Plasma C-Reactive Protein and Illusions/Hallucinations in Subjects with Parkinson’s Disease: Case–control Study
Source: PLoS One. 2014 Jan 31;9(1):e85886. doi: 10.1371/journal.pone.0085886 (PMC3908859; doi:10.1371/journal.pone.0085886)

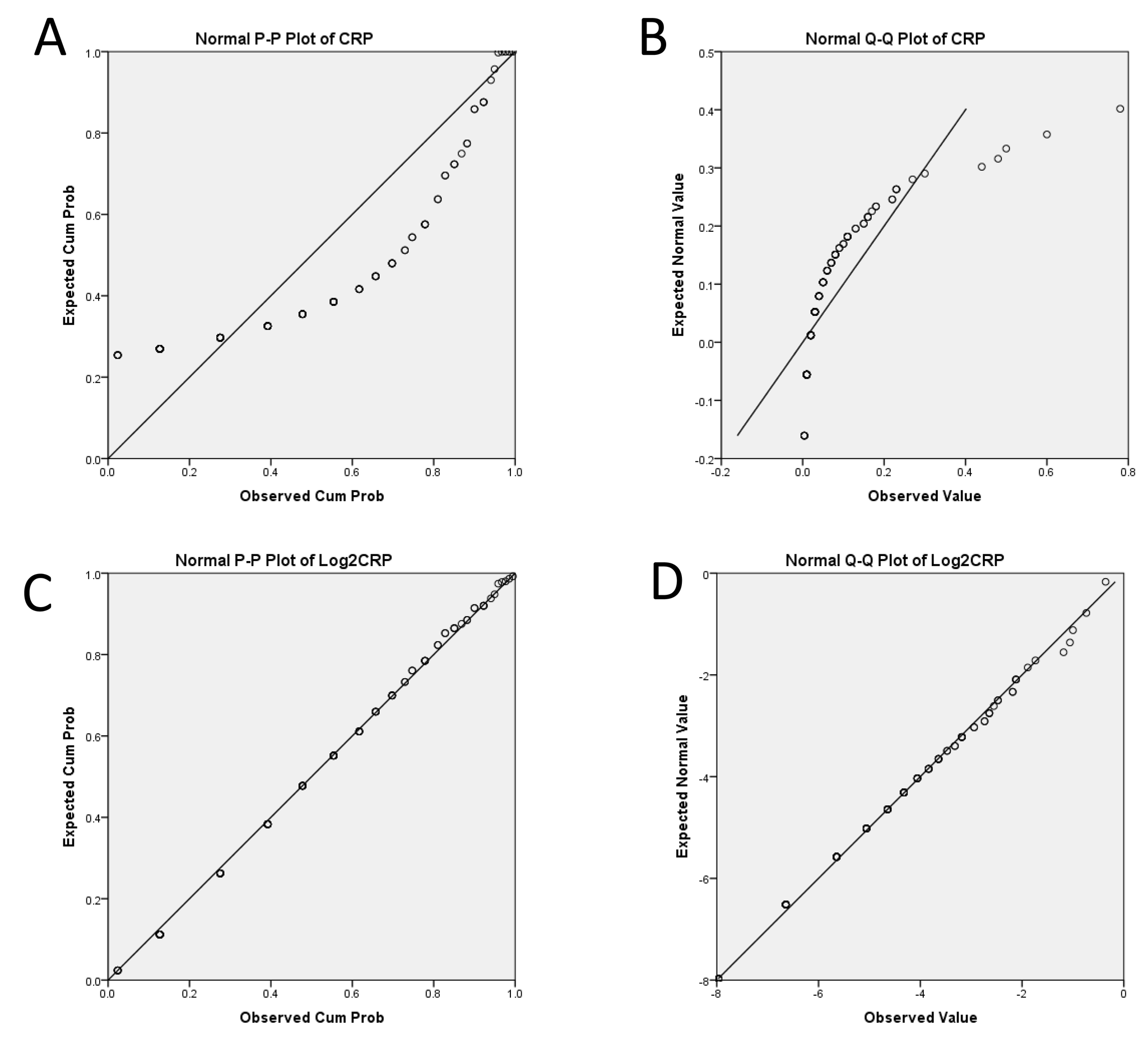

Supplement: Figure S1 — Normal P-P plots and normal Q-Q plots of plasma CRP (A, B) and log2CRP (C, D). Normal P-P plots and normal Q-Q plots of plasma CRP were shown in A and B, respectively, suggesting that the distribution of CRP was not Gaussian. In contrast, Normal P-P plots and normal Q-Q plots of log2CRP suggested that the distribution log2CRP was Gaussian (C, D). (TIF) [file pone.0085886.s001.tif]

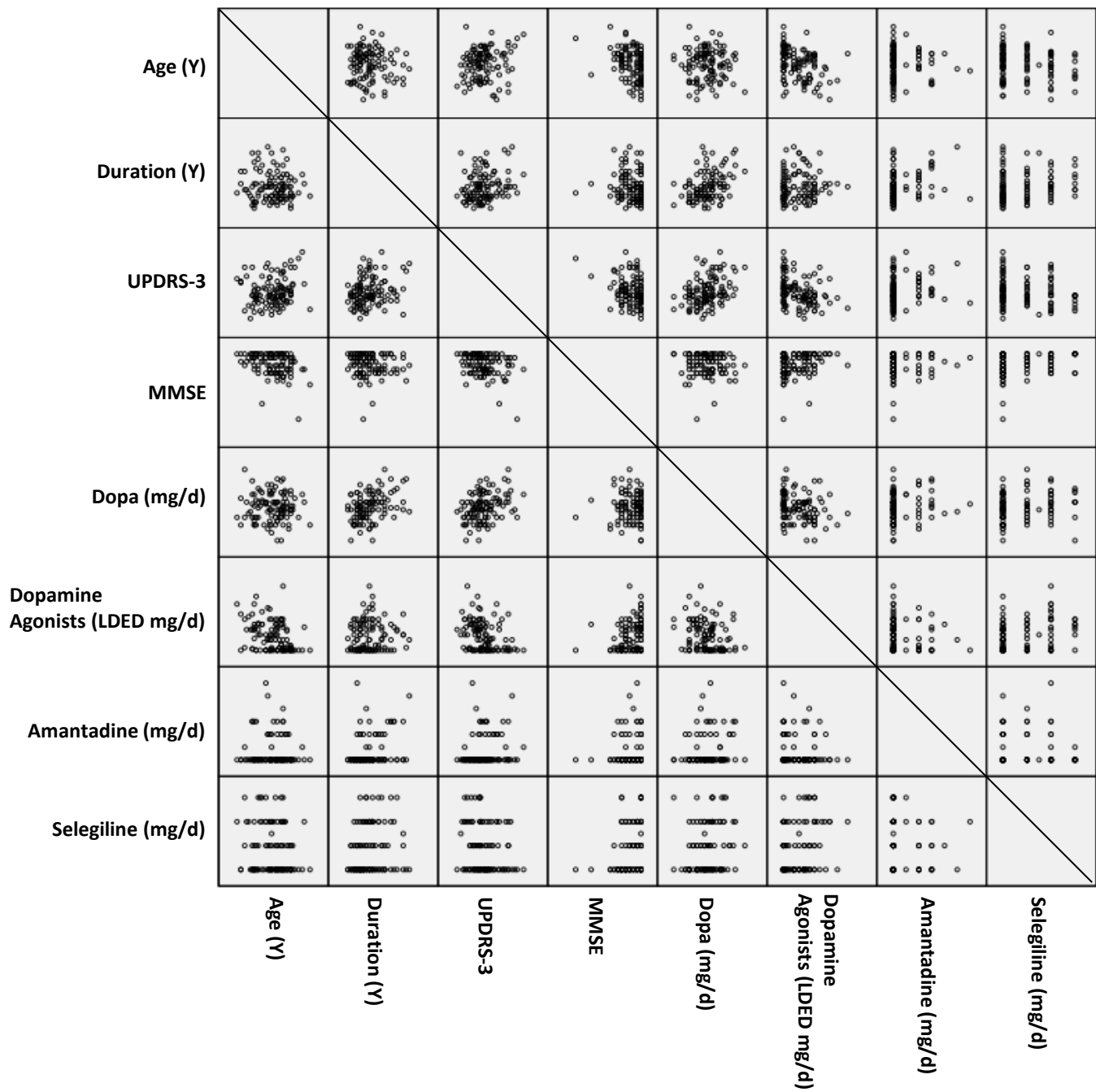

Supplement: Figure S2 — Matrix scattered plots of predictable variables. Scale variables (age, duration of PD, UPDRS-3 scores, MMSE scores, and daily dose of Dopa, dopamine agonists, amantadine, and selegiline) were plotted to check their multicollinearity. There was no multicollinearity in these scale variables. (PDF) [file pone.0085886.s002.pdf]

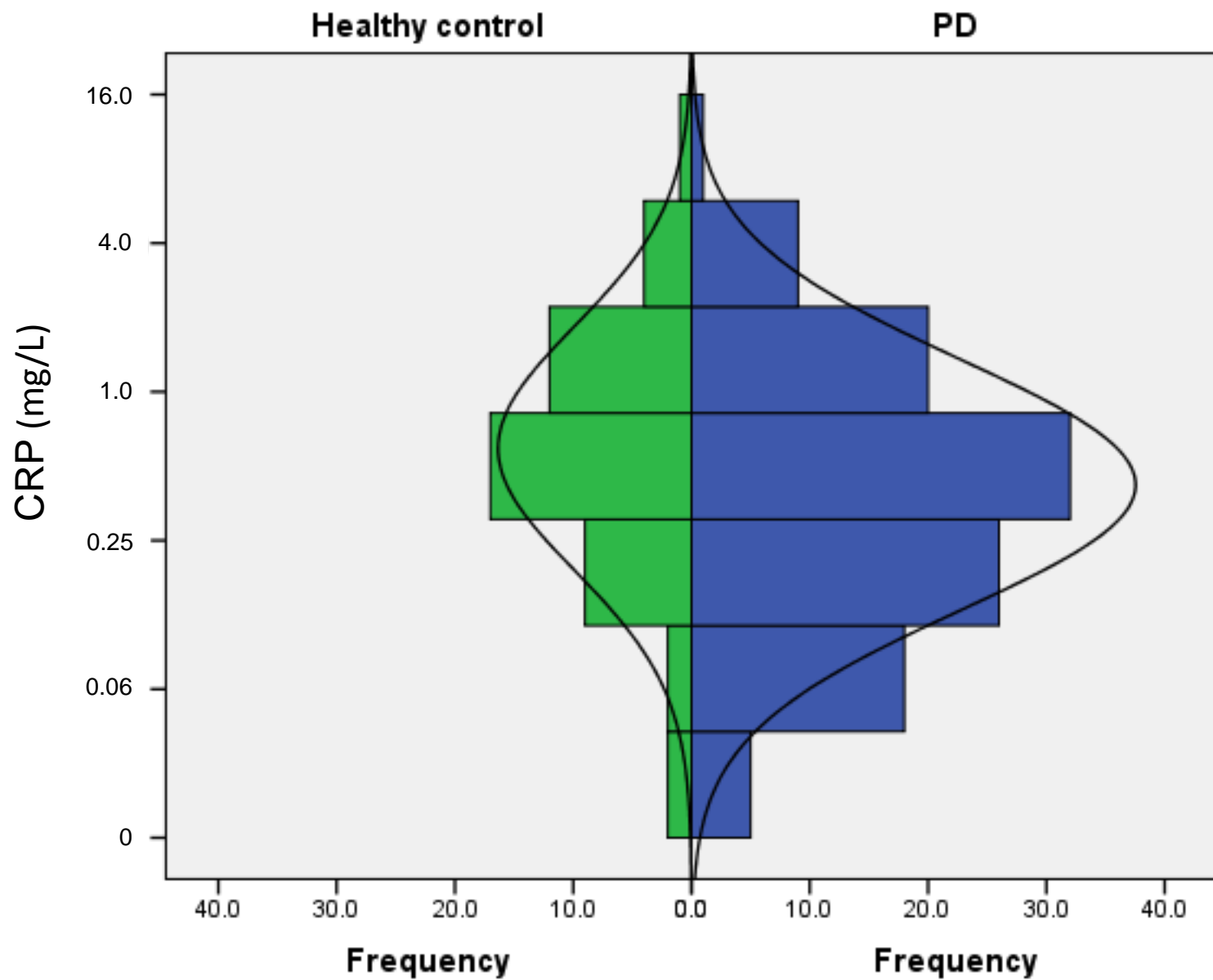

Supplement: Figure S3 — Histogram of log2CRP in PD patients and healthy controls. Log2CRP was distributed normally both in PD patients (blue, n = 111) and healthy elderly controls (n = 47, green). The distribution was very similar in PD patients and healthy controls. (PDF) [file pone.0085886.s003.pdf]
