# Supplementary material for: Subclinical Elevation of Plasma C-Reactive Protein and Illusions/Hallucinations in Subjects with Parkinson’s Disease: Case–control Study
Source: PLoS One. 2014 Jan 31;9(1):e85886. doi: 10.1371/journal.pone.0085886 (PMC3908859; doi:10.1371/journal.pone.0085886)
Supplement: Table S1 — Relationship between PPQ-B (hallucinations/illusions) and PPQ-C (delusions). (PDF) [file pone.0085886.s004.pdf]

Table S1. Relationship between PPQ-B (hallucinations/illusions) and PPQ-C (delusions)

		PPQ-C scores (delusions)				
		0	3	4	5	Total
PPQ-B scores (hallucinations/illusion)	0	83	0	0	0	83
	1	9	0	0	0	9
	2	8	0	3	1	12
	3	4	0	0	0	4
	4	0	0	1	0	1
	6	1	1	0	0	2
Total		105	1	4	1	
