# Supplementary material for: Subclinical Elevation of Plasma C-Reactive Protein and Illusions/Hallucinations in Subjects with Parkinson’s Disease: Case–control Study
Source: PLoS One. 2014 Jan 31;9(1):e85886. doi: 10.1371/journal.pone.0085886 (PMC3908859; doi:10.1371/journal.pone.0085886)
Supplement: Table S2 — Psychosis in 28 cases. (PDF) [file pone.0085886.s005.pdf]

Table S2. Psychosis in 28 cases

	n	(%)
illusions	13	(46%)
visual hallucinations	19	(68%)
auditory hallucinations	14	(50%)
tactile hallucinations	2	(7%)
delusions	6	(21%)
