# Supplementary material for: Subclinical Elevation of Plasma C-Reactive Protein and Illusions/Hallucinations in Subjects with Parkinson’s Disease: Case–control Study
Source: PLoS One. 2014 Jan 31;9(1):e85886. doi: 10.1371/journal.pone.0085886 (PMC3908859; doi:10.1371/journal.pone.0085886)
Supplement: Table S3 — Spearman’s correlation coefficient between scale predictable variables. (PDF) [file pone.0085886.s006.pdf]

Table S3. Spearman's correlation coefficent between scale predictable variables

	Age (Y)	Duration of PD (Y)	UPDRS-3 (point)	MMSE (point)	Dopa (mg/d)	Agonist (LDED, mg/d)	Aamantadine (mg/d)	Selegiline (mg/d)
Age (Y)	1.00	-0.04	0.17	-0.31	0.00	-0.37	-0.04	-0.14
Duration of PD (Y)		1.00	0.23	-0.11	0.37	0.10	0.28	0.24
UPDRS-3 (point)			1.00	-0.26	0.34	-0.36	0.22	-0.17
MMSE (point)				1.00	-0.12	0.34	0.06	0.24
Dopa (mg/d)					1.00	-0.27	0.15	0.25
Agonist (LDED, mg/d)						1.00	-0.08	0.29
Aamantadine (mg/d)							1.00	0.05
Selegiline (mg/d)								1.00
